# Gut microbiota from essential tremor patients aggravates tremors in mice

**DOI:** 10.3389/fmicb.2023.1252795

**Published:** 2023-11-23

**Authors:** Ruo-Xin Zhang, Jia-Ting Xu, Hao-Jie Zhong, Ying-Li Cai, Yu-Pei Zhuang, Ya-Ting Xie, Xing-Xiang He

**Affiliations:** Department of Gastroenterology, Research Center for Engineering Techniques of Microbiota-Targeted Therapies of Guangdong Province, The First Affiliated Hospital of Guangdong Pharmaceutical University, Guangzhou, Guangdong, China

**Keywords:** gut microbiota, washed microbiota transplantation, microbiome-gut-brain axis, tremor, essential tremor

## Abstract

**Background and objective:**

Essential tremor (ET) lacks effective treatments because its underlying mechanism is largely unknown, but may involve gut microbiota via the microbiome-gut-brain axis. We explored the effects of gut microbiota on ET in mice.

**Methods:**

Specific pathogen-free C57BL/6J mice were gavaged with stools from ET patients or matched healthy individuals. After 3 weeks of gavaging, behavioral tests were performed on all mice. Next, each mouse was injected with harmaline to induce tremors. The tremor duration was recorded; the tremor score was estimated every 30 min. Behavioral tests were repeated after modeling. Intestinal tissues and fecal samples of the mice were examined using histology and 16Sr DNA sequencing, respectively.

**Results:**

Compared with mice receiving microbiota from healthy controls, mice receiving fecal suspensions from ET patients showed worse performance in the pre-modeling behavioral tests. After modeling, ET-group mice showed significantly greater tremor scores, longer tremor duration, and worse motor performance. They also had significantly lower body weight and lower fecal pellet count. Pathological scoring revealed more severe intestinal lesions in ET-group mice. The 16S rDNA sequencing data revealed significant differences in microbiota indices, and a correlation between these indices and tremors in mice. Functional predictions indicated that the abundance of GABA-related enzymes was altered in ET-group mice.

**Conclusion:**

Mice transplanted with gut microbiota from ET patients showed worse performance in behavioral tests. After modeling, ET-group mice presented longer tremor duration, higher tremor score, and worse motor performance. This study provides evidence for gut microbiota dysbiosis that may affect the pathogenesis of ET.

## Introduction

Essential tremor (ET) is a common neurological disease characterized by bilateral action tremors of the upper limbs (Haubenberger and Hallett, 2018). The estimated prevalence of ET among people aged 65 years or older is 4.6% (Louis and Ferreira, 2010). Although the mechanism underlying ET is still debated, several evidence-based hypotheses point to different pathological causes. The γ-aminobutyric acid (GABA) hypothesis suggests that ET may associate with low levels of GABA (Málly and Baranyi, 1994) and its receptors in the cerebellum (Paris-Robidas et al., 2012). At present, β-blockers, propranolol, and primidone are used as first-line drugs for the treatment of ET (Zesiewicz et al., 2011; Zappia et al., 2013), though these treatments are not ideal. The treatment of ET is limited by a lack understanding of the disease mechanism, and new avenues of research are required to develop effective therapeutic strategies for ET.

The influence of the gut microbiota on human physiology and pathology is gradually being revealed in multiple organ systems. Gut dysbiosis has been related to gastroenterological diseases like *Clostridium difficile* infection, Crohn’s disease, and constipation (Ooijevaar et al., 2019), for which treatments such as probiotics and fecal microbiota transplantation have been proved effective. The role of the gut-brain axis has also been widely discussed in the pathogenesis of neurological diseases. The association between the gut microbiota and the central nervous system is bi-directional, as proven by a variety of evidence. Studies have found that the gut microbiota of patients with different central nervous system diseases, such as autism spectrum disorder (Palkova et al., 2021), Parkinson’s disease (Li et al., 2019), and Alzheimer’s disease (Ling et al., 2020), significantly differs from that of healthy controls. Experimental studies have found that intestinal microbiota can cause or aggravate central nervous system diseases. In animal experiments, transplantation of the intestinal microbiota from patients with neurological diseases aggravated the disease in model animals or caused healthy animals to manifest the symptoms of diseases such as Parkinson’s disease (Li et al., 2019) and multiple sclerosis (Berer et al., 2017). The specific pathways through which intestinal microbes affect the central nervous system include the immune system, tryptophan metabolism, vagus and enteric nervous system, and microbial metabolites (Cryan et al., 2019). Neuro-modulating metabolites of microorganisms, including GABA, noradrenaline, 5-hydroxy-tryptamine, and catecholamine, may play an important role in nervous system diseases (Morais et al., 2021).

The aim of our study was to determine the impact of human gut microbiota from ET patients on tremor symptoms and behavior in mouse model. We previously found that tremor symptoms were gradually relieved in an ET patient who underwent 4 courses of washed microbiota transplantation for the treatment of irritable bowel syndrome (Liu et al., 2020). This suggests a possible, yet undiscovered, link between the gut microbiota and ET. We hypothesized that the transplantation of the gut microbiota of ET patients to a mouse model of harmaline-induced tremors would aggravate the tremor symptoms and affect the behavior of the mice. To test this hypothesis, we colonized specific pathogen-free mice with microbiota collected from ET patients (henceforth called mice^ET^) or sex- and age-matched healthy controls (mice^HC^). We used behavioral tests to compare the outcomes of transplanting the different gut microbiota to the mice. After inducing the harmaline tremor models, we performed tremor scoring and repeated the behavioral tests at different time points to measure the tremor symptoms and motor performance. Histological sections of the mouse colon and small intestines were examined to grade the intestinal lesions. Differences in microbial diversity and composition were evaluated using 16S rDNA sequencing analysis of fecal samples from both groups of mice. Functional prediction of the gut microbiota was conducted to discover the potential mechanism leading to tremor symptoms and altered behavior.

## Methods

### Subject recruitment

We collected stool samples from 6 ET patients (ETd) and 6 age- and sex-matched healthy controls (HCd). We included patients who had been diagnosed with ET, and excluded those who had any other neurological diseases (such as Parkinson’s disease), organic diseases of the digestive system (such as tumor and ulcer), any other severe disease, or a history of antibiotic or probiotic use within 1 month.

Healthy controls were enrolled after ruling out the following on the basis of a detailed history: any neurological disorder, organic diseases of the digestive system, systemic medical illness, and antibiotic or probiotic use within 1 month. The detailed characteristics of the recruited subjects are shown in Supplementary Table S1.

Written informed consent was obtained from all recruited sample donors. The study was reviewed and approved by the ethics committee of the First Affiliated Hospital of Guangdong Pharmaceutical University.

### Sample collection

The fecal samples were prepared for microbiota transplantation according to an established method described previously (Liu et al., 2020; Zhang et al., 2022). Briefly, the fecal specimens were suspended in saline and filtered by an automatic intelligent fecal bacteria-separation system (GenFMTer, Nanjing, China) within 4 h of collection. We prepared a fecal suspension for each patient separately and centrifuged it at 2,500 rpm for 3 min, for a total of 3 times. After each centrifugation, the supernatant was discarded. Then, the bacterial solution was mixed with the same volume of normal saline; 50% sterile glycerol was added, and the resultant solution was aliquoted into sterile Eppendorf tubes and stored at −80°C freezer. The frozen bacterial suspensions were taken out and revived before each gavage, and glycerol was removed by centrifugation after thawing. Equal quantities of the 6 tubes of bacterial suspension in each group were mixed together, and 0.6 mL of the mixed bacterial suspension was added to 6 mL of normal saline to obtain the final microbiota suspension for transplantation.

### Experimental mice

Male C57BL/6J mice were used for the animal study, and were housed under a standard, specific pathogen-free environment. All the animal experimentation procedures were carried out according to the guidelines of the First Affiliated Hospital of Guangdong Pharmaceutical University for reporting research on animals. The animals were kept under standard conditions (12/12 light-dark cycle) with free access to food and water. Mixed antibiotics, including ampicillin (1 g/L), vancomycin (0.5 g/L), neomycin (0.5 g/L), gentamycin (100 mg/L), and erythromycin (10 mg/L), were provided in drinking water for 2 weeks to deplete the original gut microbiota (Sampson et al., 2016).

### Fecal microbiota transplantation

Approximately 48 h after the last dose of antibiotics, the mice were randomly classified into 2 groups. Each mouse received 200 μL of the microbiota suspension from patients or controls through oral gavage, three times per week for 3 weeks to reconstruct the gut microbiota of the recipient mice (Zhu et al., 2020). The body weight of each mouse was measured every week during the gavaging period.

### Fecal pellet count

The fecal output of each mouse was measured by counting its fecal pellets. The mice were put into empty cages separately, and their fecal pellets were counted every 5 min, over a cumulative 15 min period (Sampson et al., 2016).

### Pre-modeling behavioral tests

After completion of the gavaging process, all mice were subjected to the following behavioral tests in order to test their behavior and motor function.

#### Open field test

Open field tests were undertaken to measure the spontaneous movements of the mice. Each mouse was put in the middle of the open field apparatus, and its spontaneous activities in 5 min were recorded by a built-*in camera* (Vaziri et al., 2015). The total distance, average speed, and moving time were automatically analyzed by the tracking system of the apparatus (Yuyan, Shanghai, China). The test equipment was cleaned with 75% ethanol before testing the next mouse in order to remove the smell of the last animal.

### Pole test

A 50 cm-long, 1 cm-wide pole with a spherical top (diameter, 2 cm) was wrapped with gauze and placed in the cage, vertical to the ground. Mice were placed on the top of the pole facing the ground, and their time to return to the ground was recorded. Timing began when the experimenter released the animal and ended when both fore-limbs reached the base. The test was performed 3 times for each animal, and the average time was calculated for each mouse and taken as its final result (Sun et al., 2018).

#### Grip strength test

The Grip Tester for rats and mice (Zhishi Duobao, Beijing, China) was used to measure the neuromuscular strength of the mice. Experimenters held the tail of each mouse, as it gripped a metal grid by its fore limbs or hind limbs. The Grip Tester automatically displayed the grip strength in grams. Each mouse was tested 3 times, and the average result was taken (Mao et al., 2016).

#### Rotarod test

An accelerating rotarod apparatus (Xinruan, Shanghai, China) was used to measure the motor activity of the mice. All mice were trained to use the equipment for the same amount of time before the pre-modeling behavioral tests. The test was started at a speed of 10 rpm and increased up to the maximum speed of 40 rpm. Each mouse underwent 3 trials, with an inter-trial interval of 30 min. The total duration that each mouse spent on the rod maintaining its balance was taken as the final result (Vaziri et al., 2015).

### Induction of tremor models

Harmaline hydrochloride dihydrate (Sigma, Germany) was dissolved in normal saline immediately before use (Rahimi Shourmasti et al., 2012). Animal models of tremor were induced using intraperitoneal injections of harmaline solution at a concentration of 10 mg/kg body weight (Wilms et al., 1999).

### Tremor observation and scoring

The occurrence of tremors was rated by 2 observers. The timing of harmaline administration, tremor occurrence, and tremor termination was recorded. Tremor data were quantitatively scored every 30 min according to the following criteria: 0 points, no tremor; 1 point, occasional tremors affecting only the head and neck; 2 points, intermittent (occasional tremors affecting all body parts); 3 points, persistent (persistent tremors affecting all body parts and tail); and 4 points, severe (persistent tremors rendering the animal unable to stand and/or walk) (Tariq et al., 2001; Al-Deeb et al., 2002).

### Post-modeling behavioral tests

After harmaline administration, the behavioral tests were repeated to measure the motor function in the tremor models. Open field tests were implemented when the mice started to present tremor symptoms. Pole tests and grip strength tests were performed at the start of the tremors, and repeated every 1 h after the tremors had started. The average of the results at each time point was recorded. Rotarod tests were implemented at the start of the tremors, and repeated every 30 min after the tremors had started.

### Histological evaluation

After the completion of the post-modeling behavioral tests, the mice were sacrificed, and tissue samples were collected from small intestine and colon. The intestinal samples were preserved in 10% formalin for subsequent histological evaluation. The colon and small intestine tissue samples were made into 4 μm-thin paraffin sections and stained with hematoxylin and eosin. The colon sections were scored in 5 categories: inflammation, epithelial damage, edema, goblet cell loss, and mucosal hyperplasia, by using criteria from former studies (Berg et al., 1996; Ma et al., 2017). The villus height and crypt depth in the small intestine sections were measured (10 measurements per section), and the average value was taken as the final result for each section. The villus height-to-crypt depth ratio was then calculated (Clarke et al., 1976).

### Microbial DNA extraction and sequencing

Fecal samples were collected from all the mice after 3 weeks of gavage and before the induction of the harmaline models. The feces were collected in sterilized cages, frozen in liquid nitrogen for 20 min, and then stored at −80°C until analysis. The fecal samples from both groups of mice were subjected to 16S rDNA sequencing. Bacterial DNA was extracted using the CTAB/SDS method. We used 1% agarose to monitor the concentration and purity of the extracted DNA. Then, the DNA was diluted to 1 ng/μL with sterile water depending on its concentration. The 16S rDNA genes in distinct regions (V3-V4) were amplified using specific primers (341F: 5′CCTAYGGGRBGCASCAG3′, 806R: 5′-GGACTACNNGGGTATCTAAT-3′) and barcodes. The PCR mixtures were denatured at 98°C for 1 min, followed by 30 cycles at 98°C (10 s), 50°C (30 s), and 72°C (30 s), with a final extension at 72°C for 5 min. The PCR products were mixed with an equal volume of 1X loading buffer (containing SYB green) and subjected to electrophoresis on 2% agarose gels. The Qiagen Gel Extraction Kit (Qiagen, Germany) was used to purify the mixed PCR products. Sequencing libraries were generated using NEBNext^®^ Ultra^™^ IIDNA Library Prep Kit (New England Biolabs, United States). The library quality was evaluated on the Qubit@ 2.0 fluorometer (Thermo Scientific) and quantified using the Agilent Bioanalyzer 2100 system. Finally, the library was sequenced on an Illumina NovaSeq6000 platform.

### 16S rDNA gene sequencing data analysis

The reads of the samples were spliced using FLASH software (version 1.2.11) (Magoč and Salzberg, 2011) to obtain raw tags. Then, fastp software (version 0.20.0) was used to obtain clean tags. Chimera sequences were removed from the clean tags to obtain the final effective tags (Haas et al., 2011).

The effective tags were denoised using the DADA2 module (Callahan et al., 2016, 2019) in QIIME2 software (version QIIME2-202006), and sequences with an abundance of <5 were filtered out to obtain the final amplicon sequence variants (ASVs) and feature table. The obtained ASVs were compared using the Silva database to perform species annotation by using the sklearn classifier in QIIME2 (Bokulich et al., 2018; Bolyen et al., 2019). Multiple sequence alignment was performed using QIIME2 software, and then, the absolute abundance of the ASVs was normalized.

Alpha diversity indices were calculated using QIIME2, including observed operational taxonomic units (OTUs), Chao1, Shannon, Simpson, dominance, and Pielou_e. Beta diversity was calculated according to weighted and unweighted UniFrac distances in QIIME2 (Lozupone and Knight, 2005; Lozupone et al., 2007, 2011). Principal coordinate analysis (PCoA) (Minchin, 1987) was performed to visualize differences in complex multi-dimensional data between samples. Its results were displayed using the ade4 package and ggplot2 package of R software (version 2.15.3). We used the adonis (Anderson, 2001; McArdle and Anderson, 2001; Warton et al., 2012; Stat et al., 2013) and ANOSIM (Bray and Curtis, 1957; Chapman and Underwood, 1999) functions in the QIIME2 software to study the significance of the differences in microbial community structure between the 2 groups. A Venn diagram was drawn to show the common and unique ASVs among the groups. R software (version 3.5.3) was used to perform *t*-test analysis to find significantly different species at each taxonomic level. To find biomarkers, we used the LEfSe software (version 1.0) to perform linear discriminant analysis (LDA) effect size (LEfSe) analysis (Segata et al., 2011), and the LDA score threshold was set as 4. Alpha diversity indices and tremor-related parameters were used for correlation analysis. The PICRUSt2 software (version 2.1.2-b) was used for functional annotation analysis.

### Statistical analysis

GraphPad Prism version 8.0 (GraphPad, La Jolla, CA, United States) was used for data analysis and visualization. Data were first tested for normality, and the independent-samples *t*-test was used to analyze parametric data; the Welch correction was performed when the variance was not equal. The results were expressed as mean ± standard deviation. Nonparametric data were analyzed using the Mann–Whitney *U* test, and presented as median (interquartile range). *p* < 0.05 was considered statistically significant.

## Results

### Pre-modeling behavior tests

**Figure 1:**
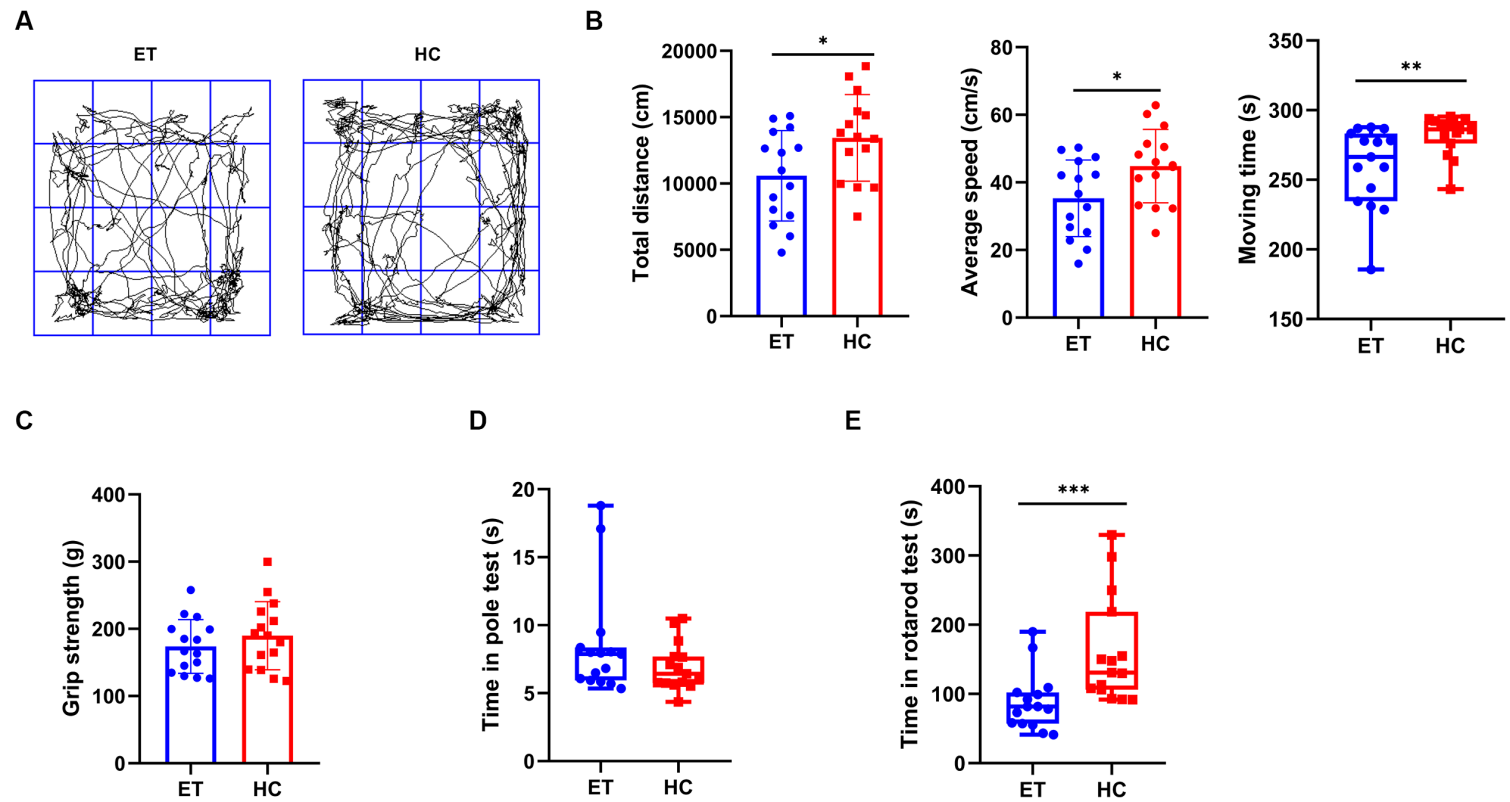
Pre-modeling behavioral tests of mice^ET^ and mice^HC^. **(A)** Representative images of the average movement path of mice in the open field test. ET, essential tremor bacteria group (*n* = 15); HC, healthy control group (*n* = 15). **(B)** Parameters in the open field test, including total distance of the mice in the open field in 5 min (mean ± standard deviation, *p* < 0.05), average moving speed of the mice in the open field (mean ± standard deviation, *p* < 0.05), and moving time of the mice in the open field (median and interquartile range, *p* < 0.05). **(C)** Pre-modeling grip strength test (median and interquartile range, *p* > 0.05). **(D)** Pre-modeling pole test (median and interquartile range, *p* > 0.05). **(E)** Pre-modeling rotarod test (mean ± standard deviation, *p* < 0.05). ^*^p < 0.05, ^**^p < 0.01, and ^***^p < 0.001.

We performed behavioral tests to discover how the different gut microbiota affected motor performance in mice (Figure 1). In the open field tests, the total distance travelled by mice^ET^ was less than that by mice^HC^ (10,995 ± 3,405 cm vs. 13,442 ± 3,261 cm, *p* < 0.05; Figure 1B, left), and their average speed was also lower (35.29 ± 11.35 cm/s vs. 44.81 ± 10.87 cm/s, *p* < 0.05; Figure 1B, middle). The median moving time of mice^ET^ was shorter than that of mice^HC^ [266.4 s (49.3 s) vs. 286.3 s (16.2 s), *p* < 0.01; Figure 1B, right]. The representative images of the average movement path of the 2 groups of mice are presented in Figure 1A. Mice^ET^ also performed worse in the rotarod tests than mice^HC^ [81.67 s (44.97 s) vs. 130.7 s (112.4 s), *p* < 0.001; Figure 1E]. The results of the pole tests and grip strength tests showed no significant difference between the 2 groups (Figures 1C,D).

### Tremor scoring and post-modeling behavioral tests

**Figure 2:**
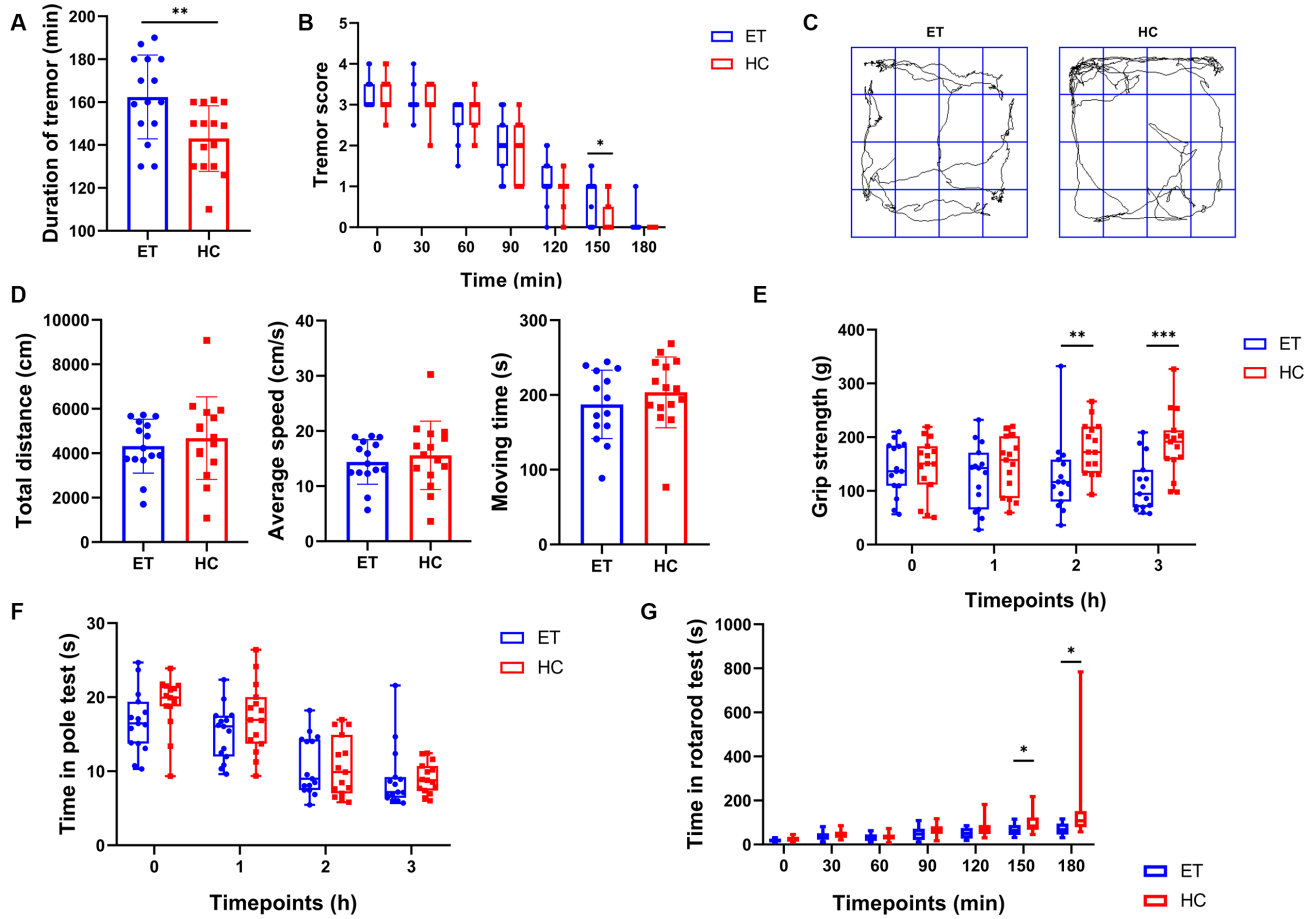
Tremor evaluation and post-modeling behavioral tests of mice^ET^ and mice^HC^. **(A)** Duration of tremor (mean ± standard deviation, *n* = 15, *p* < 0.05). **(B)** Tremor scores at each time point (median and interquartile range, *p* < 0.05 at 150 min, *n* = 15). **(C)** Representative images of the average movement trajectory in the 2 groups of mice in the open field. **(D)** Results of the open-field tests after harmaline injection, including total distance travelled in 5 min in the open field (mean ± standard deviation, *p* > 0.05), mean speed of the mice in the open field (mean ± standard deviation, *p* > 0.05), and moving time of the mice in the open field (mean ± standard deviation, *p* > 0.05). **(E–G)** Results of other behavioral tests after harmaline injection (median and interquartile range). **(E)** Grip strength test after harmaline injection (*n* = 15; *p* < 0.01 at 2 h, *p* < 0.001 at 3 h). **(F)** Pole test after harmaline injection (*n* = 15; not significant). **(G)** Rotarod test after harmaline injection (*n* = 15; *p* < 0.05 at 150 min and 180 min).

To further investigate how the gut microbiota affected tremor symptoms and motor performance in the ET models, we induced tremor in mice we used in the previous studies by injecting them with harmaline hydrochloride dihydrate. All mice exhibited tremor symptoms after the injection. Both the severity and duration of tremors were recorded. Compared to mice^HC^, mice^ET^ had a longer tremor duration (Figure 2A) and a greater tremor severity at 150 min after the commencement of tremors (Figure 2B). In the open field tests, mice^ET^ showed no difference in total distance, average speed, and moving time as compared with mice^HC^ (Figure 2D). The representative images of the average movement path is shown in Figure 2C. In the grip strength tests, the forelimb strength of mice^ET^ was significantly lower than that of mice^HC^ at 2 h after the injection [116.5 g (77.93 g) vs. 172.3 g (86.8 g), *p* < 0.01] and 3 h after the commencement of tremors [94.47 g (69.3 g) vs. 191.5 g (54.6 g), *p* < 0.001; Figure 2E]. At the other time points tested, there was a tendency for mice^ET^ to have a lower grip strength than mice^HC^, but the differences were not statistically significant. The persistence of mice^ET^ in the rotarod tests tended to be shorter than that of mice^HC^ (Figure 2G), and the difference was significant at 150 min after tremor commencement [64 s (45 s) vs. 84 s (56 s), *p* < 0.05] and highly significant at 180 min [67 s (50 s) vs. 108 s (73 s), *p* < 0.001]. No significant difference was found in the time taken in the pole tests between the 2 groups of mice at each time point (*p* > 0.05; Figure 2F).

### Body weight and fecal pellet count

**Figure 3:**
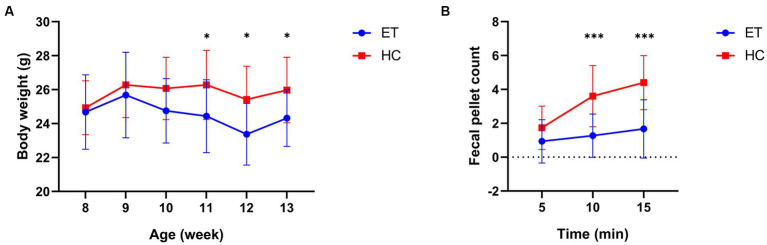
Body weight and fecal pellet count of mice^ET^ and mice^HC^. **(A)** Body weight of the mice at various age (*n* = 15, ^*^p < 0.05). **(B)** Fecal pellet count of mice over 15 min post-modeling (*n* = 15, ^***^p < 0.001).

We then analyzed the body weight and fecal pellet count of the mice. No significant difference in body weight was detected between the 2 groups before the gavage (8 weeks of age). After the gavaging had started, a significant difference was found at 11 weeks of age. The body weight of mice^ET^ was significantly lower than that of mice^HC^ at the 11th (24.44 ± 2.147 g vs. 26.28 ± 2.032 g, *p* < 0.05), 12th (23.37 ± 1.818 g vs. 25.42 ± 1.954 g, *p* < 0.05), and 13th week (24.32 ± 1.669 g vs. 25.97 ± 1.932 g, *p* < 0.05; Figure 3A).

Fecal pellet counts were conducted after the gavage to reveal any differences in bowel movements between the 2 groups of mice (Figure 3B). The fecal pellet count of mice^ET^ was significantly lower than that of mice^HC^ at 10 min (1.267 ± 1.280 vs. 3.600 ± 1.805, *p* = 0.0003) and at 15 min (1.667 ± 1.718 vs. 4.400 ± 1.595, *p* = 0.0001).

### Histological morphology analysis and scoring

**Figure 4:**
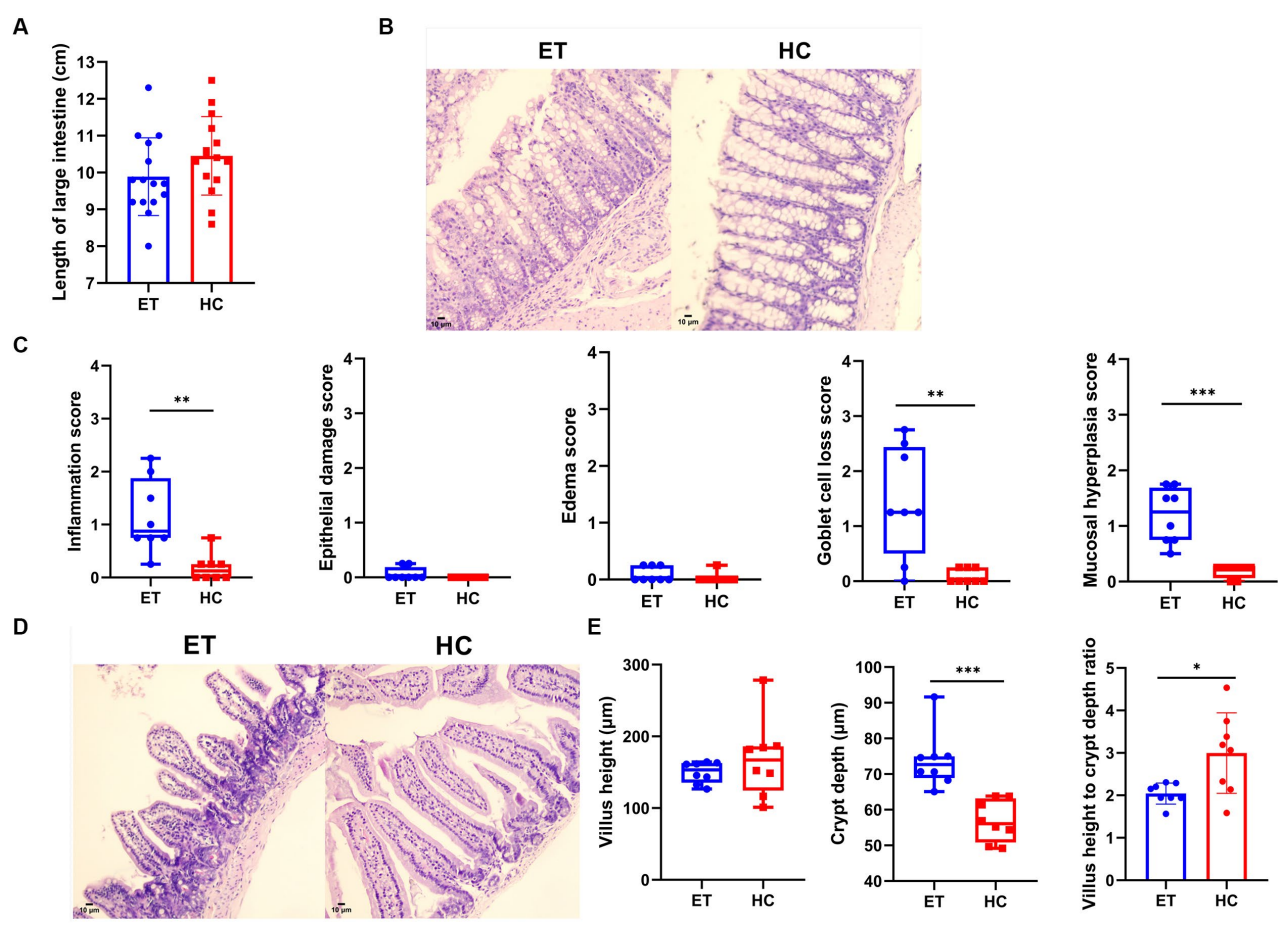
Pathological evaluation of intestines from mice^ET^ and mice^HC^. **(A)** Length of the large intestine (*n* = 15, mean ± standard deviation, ^*^p < 0.05, ^**^p < 0.01, and ^***^p < 0.001). **(B)** Pathological images of the colon (200× magnification). Compared with mice^HC^, mice^ET^ showed significant inflammatory cell infiltration and goblet cell loss. **(C)** Pathological scores of the mouse colon (*n* = 8). Normally distributed results are expressed as mean ± standard deviation, and non-normally distributed results are shown as median and interquartile range. ^*^p < 0.05, ^**^p < 0.01, and ^***^p < 0.001. **(D)** Pathological images of mouse small intestines (200× magnification). **(E)** Measurements of the small intestines. Normally distributed results are expressed as mean ± standard deviation, while non-normally distributed results are shown as median and interquartile range. ^*^p < 0.05, ^**^p < 0.01, and ^***^p < 0.001.

The length of the large intestines did not significantly differ between the 2 groups (9.887 ± 1.055 cm vs. 10.45 ± 1.064 cm, *p* > 0.05; Figure 4A). Figure 4B shows representative pathological images of the colon from both groups of mice. According to the pathological scores, mice^ET^ showed a higher degree of inflammation [0.8750 (1.125) vs. 0.1250 (0.25), *p* < 0.01], higher goblet cell loss [1.250 (1.938) vs. 0.000 (0.2500), *p* < 0.01], and a higher degree of mucosal hyperplasia [1.250 (0.938) vs. 0.2500 (0.1875), *p* < 0.001] in the colon than mice^HC^ (Figure 4B). No significant difference was found in epithelial damage [0.000 (0.1875) vs. 0.000 (0.000), *p* > 0.05] or edema [0.000 (0.2500) vs. 0.000 (0.000), *p* > 0.05; Figure 4C].

We also found differences in the pathological scores of the small intestinal tissues. The median villus height of mice^ET^ was relatively lower than that of mice^HC^, but the difference was not statistically significant [153.4 μm (27.3 μm) vs. 167.0 μm (61.6 μm), *p* > 0.05]. Representative images of the small intestines are shown in Figure 4D. The crypts were highly significantly deeper [72.67 μm (6.11 μm) vs. 52.06 μm (12.34 μm), *p* < 0.001], and the ratio of villus height to crypt depth was significantly lower (2.043 ± 0.2474 vs. 2.997 ± 0.9482, *p* < 0.05) in mice^ET^ than in mice^HC^ (Figure 4E). These results indicate that intestinal digestion and absorption were adversely affected in mice^ET^.

### 16S rDNA sequencing analysis

**Figure 5:**
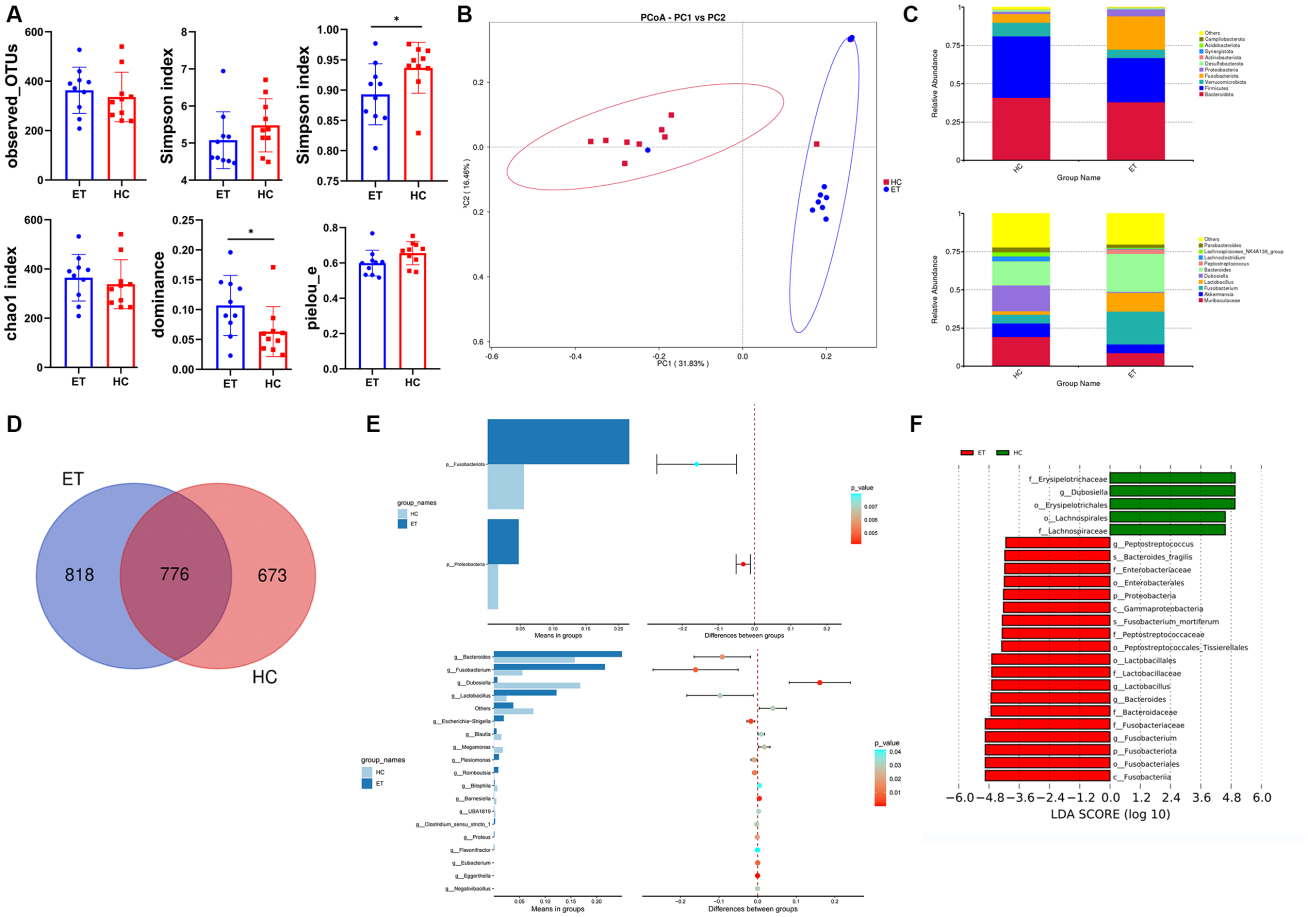
Microbial diversity and species evaluations in mice^ET^ and mice^HC^. **(A)** Alpha diversity indices of fecal samples from mice^ET^ and mice^HC^ (*n* = 10, mean ± standard deviation, ^*^p < 0.05). **(B)** Principal coordinate analysis of the abundance of microbial taxa in mice^ET^ and mice^HC^. **(C)** Stacked bar chart of species composition at the phylum level and genus level. **(D)** Venn diagram. **(E)** Differences in species between the 2 groups at the phylum level (*t*-test) and genus level (*t*-test). **(F)** Linear discriminant analysis effect size (LEfSe) analysis of the 2 groups.

Alpha diversity indices were used to reflect the intra-sample diversity (Figure 5A). The Shannon indices of fecal samples from mice^ET^ were lower than that of samples from mice^HC^, but the difference was not statistically significant (*p* > 0.05). The Simpson indices were significantly lower in mice^ET^ than in mice^HC^ (*p* < 0.05). The dominance indices of microbiota were significantly higher in mice^ET^ than in mice^HC^ (*p* < 0.05). The Pielou_e indices were relatively lower in mice^ET^ than in mice^HC^, but the difference was not statistically significant (*p* = 0.0872). The observed OTUs and Chao1 indices also did not significantly differ between the 2 groups (*p* > 0.05). Taken together, these results indicate that the diversity and evenness of microbiota were better in mice^HC^ than in mice^ET^.

Beta diversity indicates the inter-sample diversity. In the PCoA plot, samples from mice^ET^ clustered separately from those of mice^HC^ (Figure 5B). The microbial community compositions of fecal samples from mice^ET^ significantly differed from those of samples from mice^HC^ (ANOSIM: *R* > 0, *p* < 0.01).

The top 10 ASVs of each group at the phylum level and genus level are shown in stacked bar charts (Figure 5C). In this study, 818 ASVs specific to mice^ET^ and 673 ASVs specific to mice^HC^ were identified, and the Venn diagram showed 776 overlapping ASVs between mice^ET^ and mice^HC^ (Figure 5D). The *t*-test showed that Fusobacteriota (*p* < 0.01) and Proteobacteria (*p* < 0.01) had significant differences between the 2 groups at the phylum level; the relative abundances of these 2 phyla were significantly higher in mice^ET^ (*p* < 0.05; Figure 5E). At the genus level, 18 species with significant differences were recognized (*p* < 0.05; Figure 5E). LEfSe analysis was used to identify species with LDA scores >4, which indicate that the species is a biomarker with a statistically significant difference between the groups. A total of 19 biomarkers for mice^ET^ and 5 biomarkers for mice^HC^ were found (Figure 5F).

### Correlations of gut microbiota with tremor and behavior in mice

To further investigate the relationship between the gut microbiota and ET, we conducted Spearman correlation analysis to identify the correlations of alpha diversity and species abundance with tremor and behavior.

**Figure 6:**
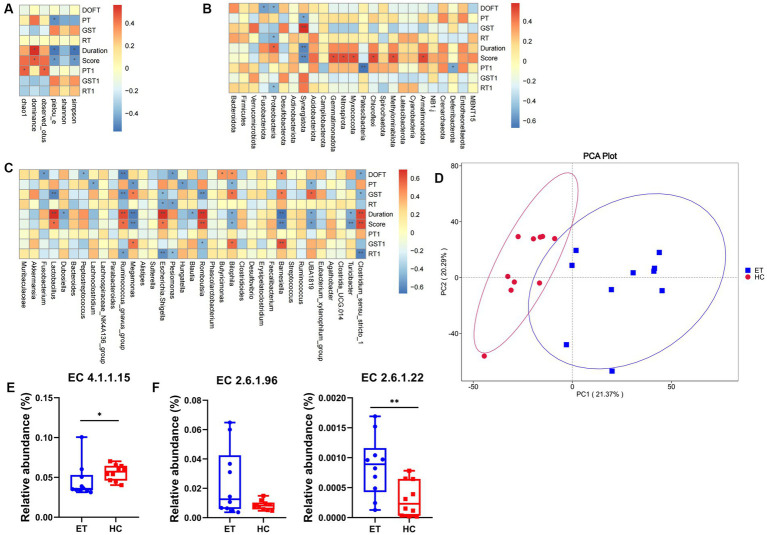
Correlation analysis and functional prediction in mice^ET^ and mice^HC^. **(A)** Spearman correlation analysis of alpha diversity with tremor and behavior. **(B)** Spearman correlation analysis of tremor and behavior with species differences at the phylum level. **(C)** Spearman correlation analysis of tremor and behavior with species differences at the genus level. **(D)** Functional annotation principal component analysis. **(E)** Predicted relative abundance of enzymes synthesizing GABA based on the EC database. **(F)** Predicted relative abundance of enzymes metabolizing GABA based on the EC database (*n* = 15, mean ± standard deviation). ^*^p < 0.05, ^**^p < 0.01, and ^***^p < 0.001. DOFT, distance in open field test; PT, pole test; GST, grip strength test; RT, rotarod test.

As illustrated in Figure 6A, Spearman correlation analysis showed that several alpha diversity indices were significantly correlated with tremor and behavior. In the pre-modeling pole tests, the climbing time was negatively correlated with the Pielou_e index (*p* < 0.05). After modeling, both tremor duration and tremor score were positively correlated with the dominance index (*p* < 0.05), and negatively correlated with the Simpson index and Pielou_e index (*p* < 0.05), indicating that mice with low microbiota diversity had higher tremor duration and score. After modeling, the pole test time was positively correlated with the Chao1 and Pielou_e indexes (*p* < 0.05).

At the phylum level, 8 species were positively correlated and 9 species were negatively correlated with tremor and behavior (*p* < 0.05; Figure 6B). At the genus level, 20 species were positively correlated (*p* < 0.05) and 34 species were negatively correlated with tremor and behavior (*p* < 0.05; Figure 6C).

### PICRUSt2 functional prediction

PICRUSt2 can be used for metagenomic function prediction based on marker genes. We performed functional prediction based on the KEGG Orthology (KO) and EC databases and 16S sequencing data. Principal component analysis was performed based on the abundance statistics of functional annotations in the KO database, a database for studying gene function. A deviation was found between the confidence ellipses of the samples from the 2 groups, suggesting a difference in the functional composition of the microbiota between the 2 groups of mice (Figure 6D).

The EC database mainly provides information on enzymes. With the background information mentioned above, the enzymes involved in the synthesis and metabolism of GABA were selected, and their relative abundances were calculated. Glutamic acid decarboxylase (GAD), which corresponds to EC4.1.1.15 in the EC database, is the key enzyme involved in the decarboxylation of glutamate to GABA. The predicted relative abundance of GAD was significantly lower in mice^ET^ than that in mice^HC^ (*p* < 0.05; Figure 6E). This may indicate a decrease in GABA concentration and an increase in glutamate concentration in mice^ET^.

4-Aminobutyrate aminotransferase (GABA-T) and L-3-aminoisobutyrate transaminase are the key enzymes involved in the metabolism of GABA, which correspond to EC2.6.1.19 and EC2.6.1.22 respectively, in the EC database. The predicted relative abundance of EC2.6.1.22 was significantly higher in mice^ET^ than in mice^HC^ (*p* < 0.01). EC2.6.1.19 showed an increasing trend, but the difference was not statistically significant (*p* = 0.051). The above result indicates that GABA metabolism might be more active in mice^ET^ (Figure 6F).

## Discussion

ET is a relatively common movement disorder. We previously reported a possible connection between ET and gut microbiota. In the present study, we found that mice transplanted with fecal samples from ET patients exhibited worse motor performance in behavioral tests. After being injected with harmaline, these mice presented more severe tremor, longer tremor duration as well as worse motor performance. Pathological scoring of the colon and small intestinal tissues confirmed that mice transplanted with feces from ET patients presented severe intestinal lesions. The results of 16S rDNA sequencing showed that fecal samples from mice^ET^ had lower microbial diversity and significantly different composition compared with samples from mice^HC^. A significant correlation was noted between microbiota indexes and tremor/behavioral indexes. Moreover, functional prediction implicated GABA-related enzymes in the pathogenesis of ET. In conclusion, we have demonstrated that gut dysbiosis plays a significant role in the occurrence and development of ET.

The novelty of our study is the use of animal models to study the potential contribution of the gut microbiota to the development and severity of ET. Extensive research has focused on the gut-brain axis and its role in neurological diseases (Sampson et al., 2016; Berer et al., 2017; Zheng et al., 2019; Zhu et al., 2020). However, the association of ET with the gut-brain axis remains unclear. One study found that ET patients had a lower Chao1 index than the negative control group and patients with Parkinson’s disease, but found no significant difference in other indices of alpha diversity; it also reported a remarkable difference in beta diversity (Zhang et al., 2022), which is consistent with our results. Shannon index of fecal samples from patients with ET (ETd) were lower than that of samples from healthy controls (HCd) (Supplementary Figure S1A). Similar findings are also noticed in fecal samples from the two groups of recipient mice (ET and HC), although the difference was not statistically significant (ETd vs. HCd: *p* > 0.05 by ANOSIM analysis), in part due to the relative small sample size. However, the difference in dominant microbes and their relative abundance at the phylum level appear to be significant in the mouse model (ET vs. HC: *p* = 0.001 by ANOSIM analysis). Overall, the fecal bacterial compositions in transplanted mice are largely reflected to those from their corresponding donors. Notably, relatively less Proteobacteria & Firmicutus but more Bacterioidota, Fusobacteriota and Verrucomicrobiota were detected in receipient mice (Supplementary Figures S1B–D). Further comparison on fecal microbiota from the two groups of recipient mice revealed that mice^ET^ had higher relative abundances of Fusobacteriota and Proteobacteria, which are both pathogenic bacteria with known roles in causing dysbiosis and abnormality of the digestive system (Shin et al., 2015; Sears, 2018) and remain unclear in ET. It could be concluded from our study and previous reports confirm that ET patients may have less diverse and distinctly altered gut microbiota (Pan et al., 2022; Zhang et al., 2022), and these alterations could be transferred to mice through FMT. In addition, we found that mice^ET^ had a lower fecal pellet count, indicating impaired intestinal function and more severe pathological lesions. Our data suggest that the different microbial diversity and composition of the gut microbiota in mice^ET^ played an important role in the functional and pathological changes in the intestines, and eventually contributed to the pathogenesis and severity of ET, although the mechanisms between these altered gut microbiota and ET remain to be elucidated.

Studies have proved that the gut microbiota can affect behavioral performance by altering the levels of GABA and GABA receptors. For example, *Lactobacillus rhamnosus* has been proved to produce GABA and regulate GABA receptors in the brain, and could attenuate depression and anxiety-like behavior in mice (Bravo et al., 2011). Studies on other neurological diseases have also demonstrated that behavioral changes might be elicited through the glutamate-glutamine-GABA cycle (Olson et al., 2018; Zheng et al., 2019). Glutamic acid can be catalyzed by GAD to form GABA, and glutamate can be catalyzed by glutamine synthetase to form glutamine. Bacteria that can synthesize GABA exist in human gut microbiota (Barrett et al., 2012; Strandwitz et al., 2019). Some bacteria can encode GAD, and some bacteria can synthesize GABA by regulating GAD. It has been proven that abnormal levels of the inhibitory neurotransmitter GABA and the excitatory neurotransmitter glutamate can affect the function of the central nervous system and behavior. A study found that germ-free mice transplanted with the gut microbiota of patients with schizophrenia developed schizophrenia-relevant behavior as well as lower glutamate levels but higher glutamine and GABA levels in the hippocampus (Zheng et al., 2019). These results suggest that the gut microbiota of schizophrenic patients can worsen the behavior of mice, possibly by affecting the levels of neurotransmitters in their hippocampus. Another study focusing on refractory epilepsy discovered that gut microbiota could modulate host metabolism and seizure susceptibility in mice; reductions in systemic gamma-glutamylated amino acids and elevated hippocampal GABA: glutamate levels correlated with seizure protection (Olson et al., 2018). In both studies, the gut microbiota may have affected the behaviors and symptoms of mice through the glutamine-glutamate-GABA cycle, which is consistent with our results.

Some limitations of this study must be acknowledged. First, we used 6 donors each for the ET and HC groups, which may not represent the overall gut microbiota of all patients with ET. Second, although the harmaline model used in this study is the conventional model of ET, it differs from the chronic tremors seen in patients. The duration of the harmaline model rarely exceeds 3 h after drug injection. It is thus impossible to make animal models before transplanting different gut microbiota or to observe long-term tremor symptoms. Instead, we administered the different microbiota to the 2 groups of mice first, and then observed whether there was any difference in tremors and motor performance after modeling. Third, tremor evaluation was performed using previously reported scoring criteria; however, the scoring results reported by different observers are not completely consistent because the animals were in a state of continuous tremor. Transgenic mouse models could be used, and a more precise evaluating system could be devised for future studies. Finally, the mechanism via which the gut microbiota affected the occurrence of ET was not deeply explored. The effects of the main contributing bacterial strains and their metabolites were not verified, and their role in ET still needs to be studied. Although our study showed multiple differences to be caused by gut microbiota, the mechanisms involved require more rigorous investigation.

In conclusion, we found that transplanting the gut microbiota from ET patients into mice induced severe tremors and worsened behavioral performance in mice. The possible causes include more severe intestinal lesions, lower gut microbiota diversity, and variation in the predicted enrichment of GABA-related enzymes. These findings suggest a crucial connection between ET and the gut microbiota, potentially providing a new diagnostic and therapeutic target for the management of ET in the future.

## Data availability statement

The data presented in the study are deposited in the NBCI repository, accession number PRJNA993863.

## Ethics statement

The studies involving humans were approved by the Ethics Committee of the First Affiliated Hospital of Guangdong Pharmaceutical University. The studies were conducted in accordance with the local legislation and institutional requirements. The participants provided their written informed consent to participate in this study. The animal study was approved by the Animal Experimentation Ethics Committee of Guangdong Pharmaceutical University. The study was conducted in accordance with the local legislation and institutional requirements.

## Author contributions

X-XH designed the study and acquired funding. R-XZ, J-TX, H-JZ, Y-LC, Y-PZ, and Y-TX performed the experiments. R-XZ analyzed the data and wrote the manuscript. All authors contributed to the article and approved the submitted version.
