# Supplementary material for: Gut microbiota from essential tremor patients aggravates tremors in mice
Source: Front Microbiol. 2023 Nov 23;14:1252795. doi: 10.3389/fmicb.2023.1252795 (PMC10705759; doi:10.3389/fmicb.2023.1252795)
Supplement: Supplementary file 1 [file Data_Sheet_1.PDF]

Gut Microbiota from Essential Tremor Patients Aggravates Tremors in Mouse Animal Model

SUPPLEMENTAL MATERIALS

Table S1. Characteristics of patients with ET and healthy controls

Age of patients with ET (years)	Sex of patients with ET	Age of their matched healthy controls (years)	Sex of their matched healthy controls
60	Male	55	Male
22	Male	23	Male
53	Female	57	Female
64	Male	66	Male
35	Male	31	Male
26	Male	25	Male

ET, essential tremor.

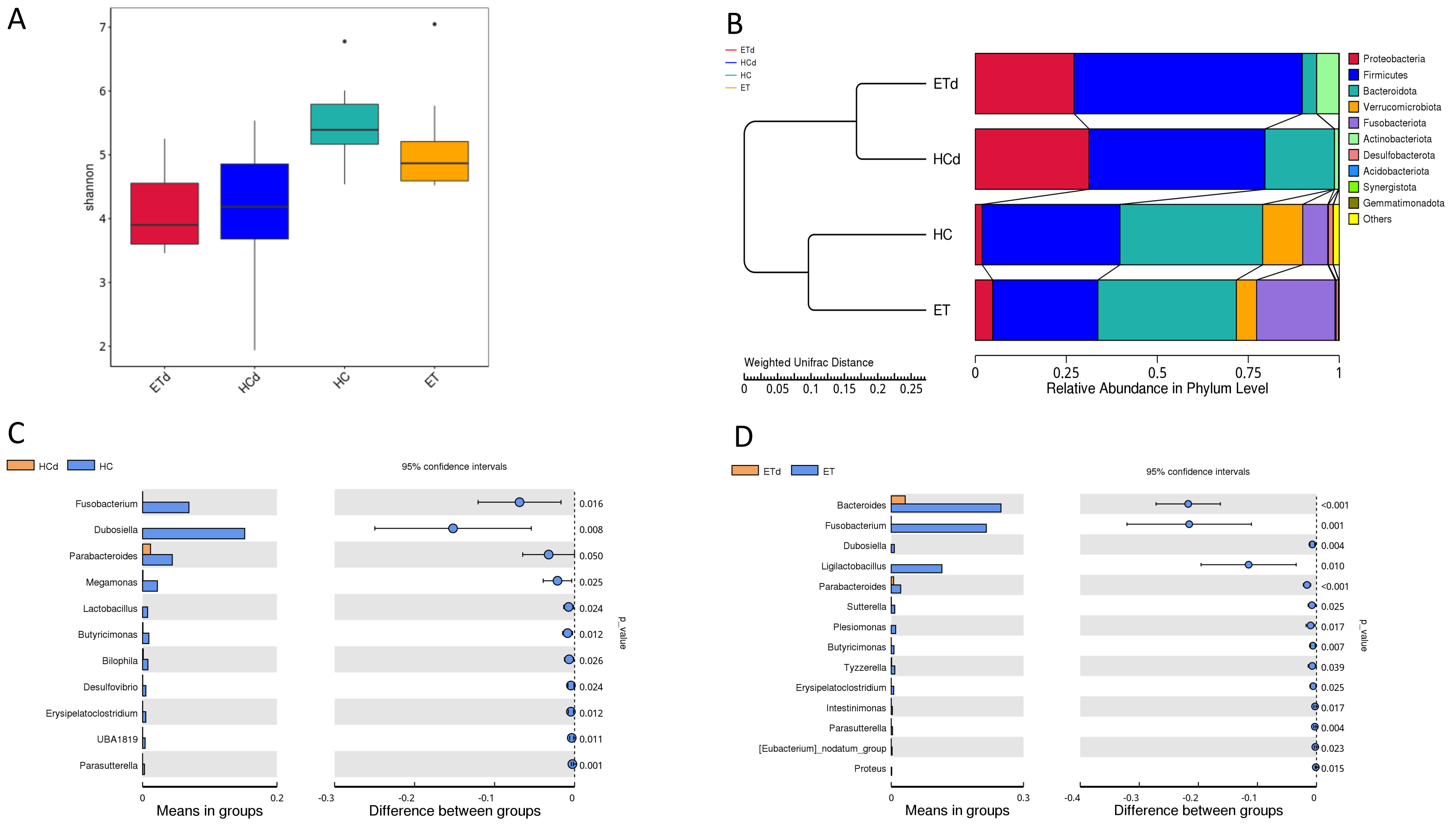

Figure S1. Comparative microbiota from human fecal transplant suspension of patients with ET (ETd), healthy control (HCd) and fecal microbiota from ET and HC groups of mice. **(A)** Alpha diversity of fecal samples from humans (ETd and HCd) and recipient mice (ET and HC). **(B)** Relative abundance of microorganisms in phylum level. **(C)** Difference in fecal microbiota between healthy human donor and mice recipients. **(D)** Difference in fecal microbiota between patients with ET and mice recipients. Refer to Figure 5 for difference in fecal microbiota between the ET and HC groups of recipient mice.
